# Impact of thermal and biotechnological processing on the bioaccessibility and allergenic peptide profile of white lupin (*Lupinus albus)*

**DOI:** 10.3389/fnut.2025.1757989

**Published:** 2026-01-28

**Authors:** Fatmanur Ozyurek-Arpa, Mustafa Yaman, Bilal Çakır, Indrani Kalkan

**Affiliations:** 1 Department of Nutrition and Dietetics, Faculty of Health Sciences, Istanbul Medipol University, Istanbul, Türkiye; 2 Department of Nutrition and Dietetics, Institute of Health Sciences, Istanbul Medipol University, Istanbul, Türkiye; 3 Department of Molecular Biology and Genetics, Faculty of Engineering and Natural Sciences, Istanbul Sabahattin Zaim University, Istanbul, Türkiye; 4 Halal Food R&D Center, Sabri Ülker R&D Center Bldg, İstanbul S. Zaim University (İZÜ), Istanbul, Türkiye; 5 IZÜ Food and Agricultural Research Center (GTUAM), Halkalı Campus, Istanbul, Türkiye

**Keywords:** allergenicity, bioaccessibility, *in vitro* digestion, *Lupinus albus*, peptide, sequence

## Abstract

**Background:**

White lupin (*Lupinus albus*) is increasingly recognized as a sustainable, highprotein functional food ingredient, yet its potential allergenicity remains a significant concern for food safety. This study aimed to investigate the impact of thermal and biotechnological processing methods on the bioaccessibility and allergenic peptide profiles of white lupin seeds.

**Methods:**

Local lupin seeds were subjected to four distinct processing techniques: boiling, microwave cooking, fermentation, and enzymatic treatment. To evaluate protein stability and allergen release under physiological conditions, the processed samples underwent simulated *in vitro* gastrointestinal digestion as well as standard protease hydrolysis. The resulting peptide sequences were characterized using liquid chromatography with tandem mass spectrometry (LC-MS/MS) and analyzed via *in silico* bioinformatics tools to predict potential allergenicity.

**Results:**

The results indicated that biotechnological processes, specifically enzymatic treatment and fermentation, generated a higher diversity of detectable peptides and distinct allergenic matches following digestion compared to thermal treatments alone. Furthermore, samples subjected to the simulated gastrointestinal digestion model revealed a broader spectrum of conserved epitope matches in allergen databases compared to standard hydrolysis, suggesting that this model provides a more accurate representation of the allergenic peptides likely to persist in the human digestive tract.

**Discussion:**

These findings demonstrate that processing methods significantly modulate the structural integrity and predicted allergenic profile of lupin proteins, providing a molecular basis for selecting appropriate processing strategies in the development of safer lupin-based functional foods.

## 1 Introduction

The global food system faces a critical challenge, as it is widely acknowledged to be unsustainable, leading to major environmental degradation and an inability to adequately nourish the global population (1, 2). This necessity drives a global transformation toward sustainable food systems (3). In recent years, this has led to a worldwide shift from animal proteins to plant-based proteins, particularly in Western countries. The primary reasons include the prominence of the health benefits of replacing animal protein with plant-based protein, the rise in the number of people adopting vegan and vegetarian diets, and the growing awareness of sustainable agriculture (4).

Lupins have garnered global attention for their high nutritional value, non-GMO status in commercial cultivation, cost-effectiveness, and superior sustainability compared to conventional crops. Lupins can fix nitrogen in the soil, absorb phosphate from the soil, and can grow in many climates. These characteristics make lupins a highly sustainable crop (5). Especially lupin proteins and peptides have been linked to various health benefits, including cell regeneration, glycemic regulation, and cholesterol reduction, primarily due to their high fiber and mineral content (6). Despite this potential, the increasing integration of lupin into processed foods presents a growing public health concern due to its allergenic potential and cross-reactivity with peanuts (7). A study confirmed the presence of lupine in 4.12–22.9% of processed food products (8). The prevalence of lupin allergy in the general population is unknown. Lupin allergens are primarily globulin storage proteins (conglutins). Conglutin b and conglutin α fraction proteins are the main lupin allergenic proteins (9).

Thermal processing of food proteins may induce structural changes such as peptide bond hydrolysis, denaturation, aggregation via disulfide or non-covalent bonds, and interactions with lipids or carbohydrates. These modifications can alter the integrity of IgE-binding epitopes, potentially increasing (via exposure or neoepitope formation) or decreasing (via epitope loss) allergenicity (10). Food processing affects the structural and allergenic properties of allergens by altering their stability and other physicochemical properties. Different processes can lead to variable changes in protein structure. Consequently, it has been established that processes can affect allergenicity in unforeseen manners (11). Recent research has demonstrated that fermentation reduces the density of tryptic peptides derived from major lupin allergens, particularly β-conglutin, suggesting a potential strategy to mitigate allergenicity in lupin-based foods. A greater reduction was noted for the major white lupin allergen based on β-conglutin peptide density. Therefore, fermentation may be beneficial for reducing the potential allergenicity of lupin-based foods (12). Few studies have investigated the effects of incorporating allergenic food ingredients into processed food matrices. Such studies are based on the hypothesis that allergenicity can be influenced by altering the progressive release of allergens from the food matrix and their susceptibility to digestion. This, in turn, affects their accessibility to the intestinal epithelium, a property referred to as “bioaccessibility.” This provides a suitable environment for gastrointestinal and intestinal absorption. The bioaccessibility of allergens can influence how they sensitize or elicit allergic reactions. For this purpose, the use of *in vitro* digestion models, which allow the evaluation of the effects of the food matrix on allergen bioaccessibility, is increasing (13).

Therefore this study aims to systematically investigate and assess peptide sequences in allergenic proteins of domestically grown *Lupinus albus* seeds subjected to various cooking methods on bioaccessibility and allergenic peptide profiles. Additionally, it seeks to address the current gap in the literature concerning the impact of *in vitro* gastrointestinal digestion on allergenic peptides. To the best of our knowledge, this is the first comparative study evaluating how different cooking methods influence the *in vitro* digestibility and bioaccessibility of lupin-derived peptides for comparison of the physiological INFOGEST model against a non-physiological, simultaneous enzymatic degradation, a standard protein hydrolysis method.

## 2 Materials and methods

### 2.1 Sample supply and transportation

White lupin (*Lupinus albus*) seeds from the (intentionally left blank for review), were supplied by a local vendor in sealed plastic bags. Prior to cooking, the seeds were stored in a light-resistant container at room temperature (22–24 °C) in a dry environment. The dried seeds were sorted and cleaned three times to remove foreign material as well as immature, and damaged seeds. Only visually intact and uniform seeds were included to ensure sample standardization. Cooking procedures were performed in a domestic setting, and the prepared samples were subsequently stored at +4 °C. To remove naturally occurring alkaloids, which may hinder widespread consumption and impart a bitter taste, thereby simulating traditional domestic preparation methods, the seeds were soaked in tap water for 5 days, with the water replaced every 12 h. Sodium bicarbonate (NaHCO_3_, 10 g/L) and sodium chloride (NaCl, 10 g/L) were added to the soaking water (14, 15). This initial soaking step, essential for making the lupin seeds palatable and safe for human consumption, is primarily an alkaloid removal process and is not expected to significantly alter the major protein structure. Subsequently different cooking techniques were applied. Cooked lupine samples were placed in airtight, transparent glass containers and transported to the laboratory in insulated boxes with ice packs (16, 17). The following cooking methods were applied to the *L. albus* samples (18).

### 2.2 Application of cooking methods

**FIGURE 1:**
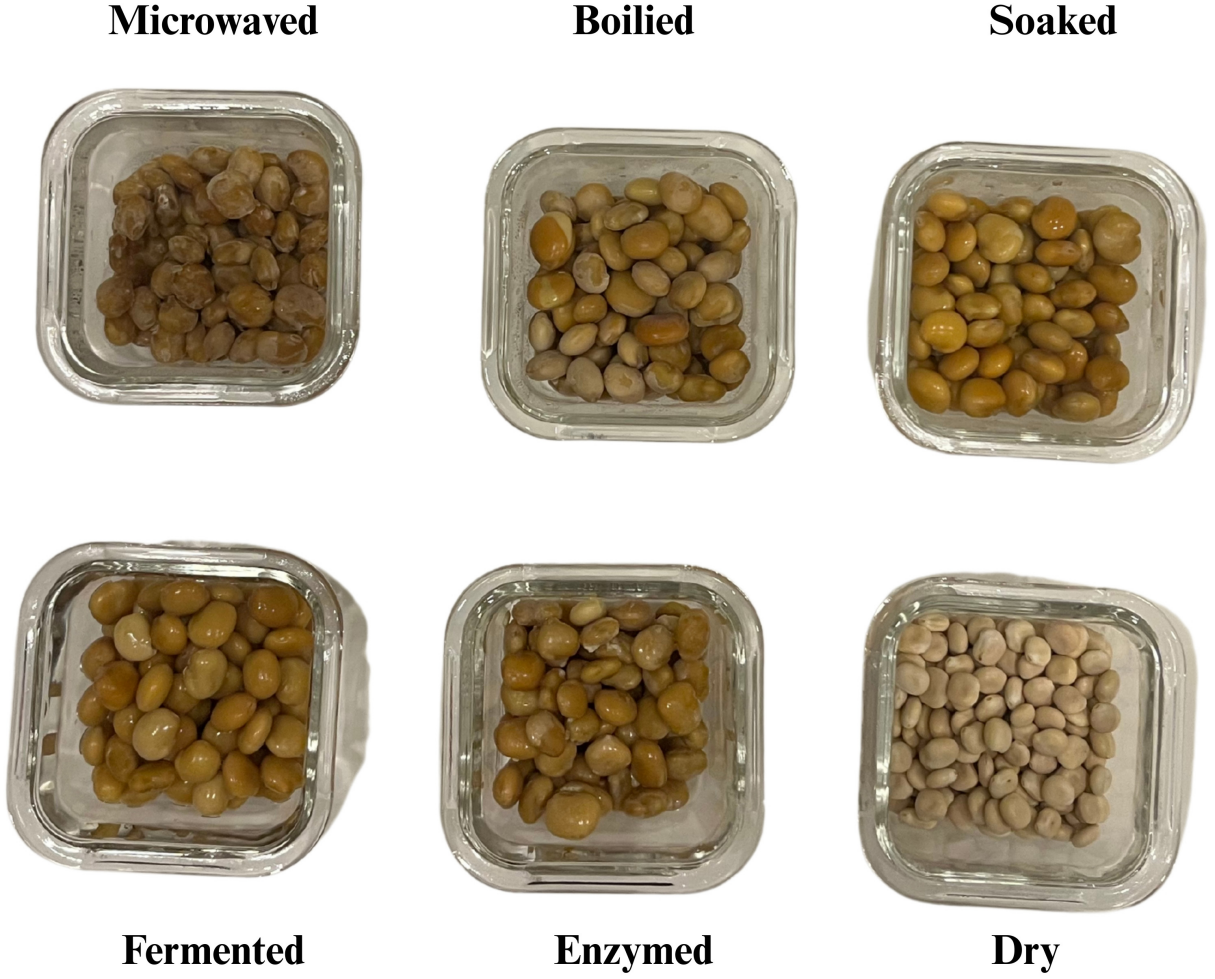
Cooked samples.

In this study, specific processing conditions were selected to represent typical household practices and standard biotechnological protocols. The aim was to compare the fundamental effects of these distinct processing mechanisms on the allergenic profile of lupin, rather than to optimize individual processing parameters (e.g., time-temperature combinations). Boiling: Samples were cooked in boiling water at 100 °C for 60 min under controlled conditions, with time and temperature monitored using a thermometer. At the end of the process, cooking was halted by rinsing the samples with ice-cold water. Microwaving: Soaked lupin samples were placed in a glass container, covered with plastic wrap, and microwaved at 900 W for 30 min, with inspection at 10-min intervals. Fermenting: Soaked lupin samples were boiled in water at 100 °C for 30 min. Subsequently, 50 g of boiled lupins were added to 200 mL of hot water containing 5 g of rock salt. Lactic acid fermentation was then performed using *Lactobacillus brevis* in a whey-based culture medium at 43 °C for 24 h in the dark, protected from sunlight (19). Enzyming: The soaked lupin samples were boiled in water at 100 °C for 30 min, then subjected to treatment with 5 g of a commercial powdered bromelain preparation (800 GDU) (20, 21). Samples were periodically tested for softness by gentle manual pressure. Cooking was considered complete, and samples were placed in storage, once a uniformly soft texture was achieved (22). Figure 1 shows images of the cooked samples.

### 2.3 *In vitro* digestibility process for lupin samples

**FIGURE 2:**
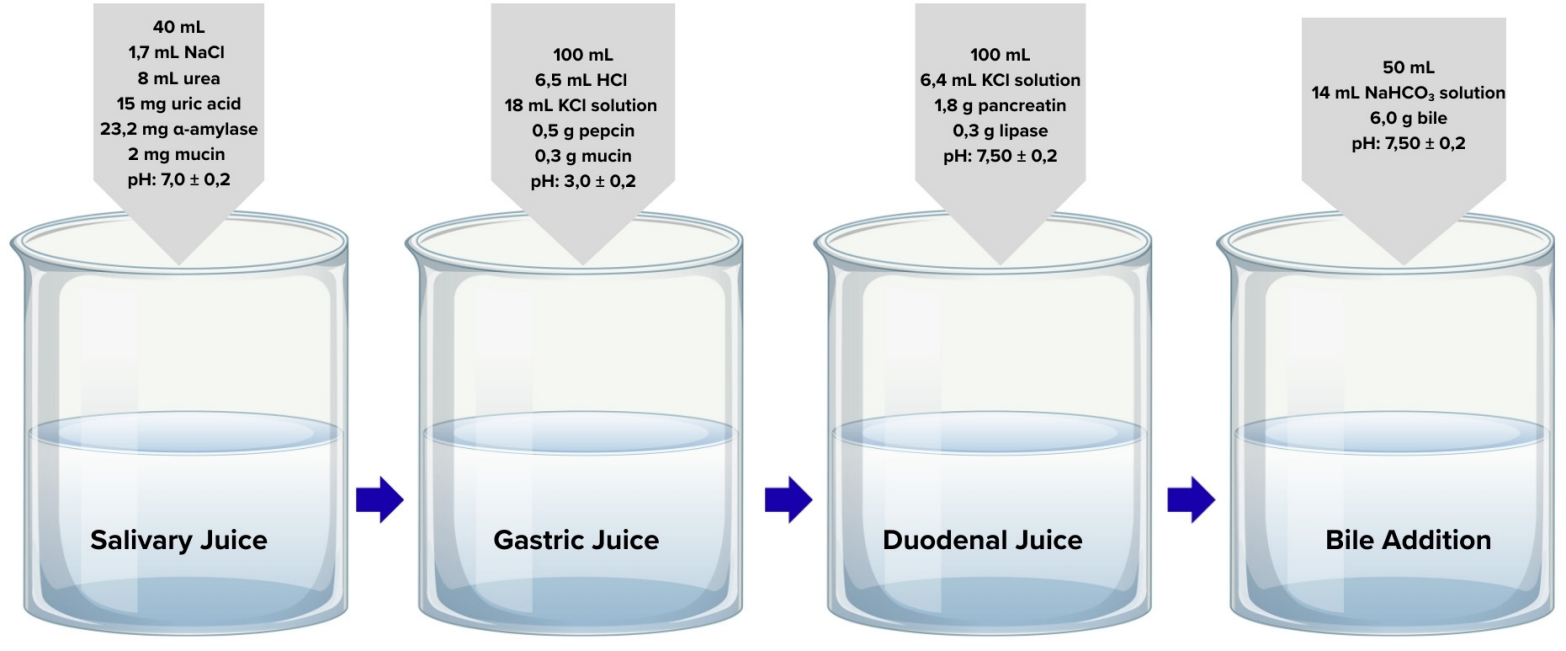
Simulated gastrointestinal digestion phases with relevant enzyme compositions and pH conditions.

A laboratory-based system was established using organic and inorganic chemicals and enzymes to simulate the mouth, stomach, and small intestine phases of the human digestive process. The procedure was based on a modified version of the INFOGEST protocol, as described by Yaman and Mızrak (23, 24). Enzyme solutions were prepared based on the specific activities provided by the manufacturer to achieve the required enzymatic activities in the final digestion mixtures. Samples were numbered, and in parallel, a blank solution without any sample was processed through all experimental steps. After each measurement, the pH meter probe and the magnetic stir bar used for mixing were rinsed thoroughly with distilled water. During intervals between experiments, mixtures in the beakers were covered with Parafilm and stored at +4 °C. A schematic representation of the digestion steps is provided in Figure 2.

For the oral phase, 2 g of sample was placed into a 10 mL Falcon tube and 2 mL of simulated saliva solution containing α-amylase from porcine pancreas (Sigma A3176, Type VI-B, ≥ 500 units/mg protein) was added. The volume was adjusted to 10 mL with distilled water, and the mixture was homogenized for 20 s. Samples were then incubated for 5 min at 37 °C in a shaking water bath, in sealed tubes (pH 7.0 ± 0.2) (23). For the gastric phase, 4.8 mL of simulated gastric solution containing pepsin (Sigma P7012, derived from porcine gastric mucosa, activity ≥ 2,500 units/mg protein) was added to the mixture from the oral phase. The mixture was homogenized for 20 s and incubated for 2 h at 37 °C in a shaking water bath, in sealed tubes (pH 3.0 ± 0.2). For the intestinal phase, 4 mL of simulated intestinal fluid containing pancreatin (Sigma P7545, from porcine pancreas, 8 × USP specifications). Based on the manufacturer’s specification (equivalent to ≥ 200 USP protease units/mg), the pancreatin solution was standardized to achieve a trypsin activity of 100 U/mL in the final digestion mixture and 2 mL of bile solution containing bile salts (Sigma B8631, bovine) were added to the mixture from the gastric phase. The samples were incubated in a shaking water bath at 37 °C for 2 h (pH 7.50 ± 0.2) (25, 26).

### 2.4 Standard proteolytic hydrolysis process for lupin (*L. albus*) sample (technical control)

To compare the physiological INFOGEST model against a non-physiological, simultaneous enzymatic degradation, a standard protein hydrolysis method was applied as described (27). It should be noted that this method was not intended to simulate human digestion but served as a technical control representing an aggressive, single-step enzymatic hydrolysis often used in proteomic sample preparation. The enzyme solutions were prepared in 100 mM Tris-HCl buffer at pH 8.0. Given that 100 g of lupin contains approximately 38 g of protein, each 2 g sample contained about 0.76 g of protein. Two grams of sample were weighed and transferred into 10 mL falcon tubes. Pepsin (enzyme-to-substrate ratio 1:200; 152 mg) (28); chymotrypsin (1:100; 76 mg); and trypsin (1:100; 76 mg) (29) enzymes were dissolved in 50 mL distilled water. Although pepsin is known to be inactive at alkaline pH, it was included in the mixture to strictly adhere to the reference protocol (27) and to represent a total protease challenge. The solution was mixed using a magnetic stirrer. One Falcon tube was designated as a “negative control” containing no sample. Five milliliters of the prepared enzyme solution were added to each tube, and the mixtures were incubated in a shaking water bath at 37 °C for 2 h (pH 7.50 ± 0.2). To denature the proteolytic enzymes in the digested solution, the pH was lowered to 3.0 using 0.1 M trichloroacetic acid, and the volume was adjusted to 50 mL with distilled water. The digested samples were centrifuged at 4,000 rpm for 5 min, and the supernatants were filtered through a 0.22 μm cellulose acetate (CA) filter. The filtrates were stored at −20 °C until analysis.

### 2.5 Lupine (*L. albus*) samples LC-MS/MS method

Peptide analyses were performed using a Waters Acquity UPLC I-Class Plus system coupled to a Xevo TQ-XS triple quadrupole mass spectrometer (Waters, Milford, MA, USA), equipped with a BEH C18 column (2.1 mm × 50 mm, 1.7 μm particle size). The mobile phase constitutes the liquid passing through the column in the LC section. The mobile phases consisted of: phase A, 0.1% formic acid in water; and phase B, 0.1% formic acid in acetonitrile. The LC gradient program is presented in Table 1.

**TABLE 1:** Gradient elution program.

Time (min)	Mobile phase B (%)	Mobile phase A (%)
0–5	15%	85%
5–8	25%	75%
8–20	50%	50%
20–32	85%	15%
32–40	15%	85%

### 2.6 LC system parameters

To ensure stable and controlled elution of peptides on the column, the column was maintained at 40 °C and the sample tray temperature at 20 °C. The injection volume was 5 μL, and the mobile phase flow rate was 0.5 mL/min. Mass spectrometric detection was performed using electrospray ionization (ESI) in both multiple reaction monitoring (MRM) and full-scan modes. The capillary voltage was set to 0.5 kV, the cone voltage to 70 eV, and the collision energy ranged from 20 to 40 eV.

### 2.7 *In silico* analyses

*In silico* analysis was used to predict the peptides generated after digestion, to verify the accuracy of LC-MS/MS results, and to compare these peptides with known allergenic protein sequences in established databases.

### 2.8 Determination of protein sequences

Analyses were performed by importing protein sequences obtained from publicly available databases into the software. Protein analysis was simulated using single, double, or triple enzyme combinations. To model gastric and small intestinal digestion of lupin proteins, two enzyme combinations were used: (i) pepsin (pH > 2; EC 3.4.23.1) + pancreatin, and (ii) pepsin (pH > 2; EC 3.4.23.1) + trypsin (EC 3.4.21.4) + chymotrypsin (EC 3.4.21.1). The ExPASy PeptideCutter tool, available through the Swiss Bioinformatics Resource Portal, was used to perform virtual enzymatic digestion of protein sequences and generate peptide fragments of varying lengths for further analysis (30). Peptide sequences were expressed using the standard single-letter amino acid code (IUPAC) following database comparisons.

### 2.9 Data optimization

Protein mass spectral data were processed using MassLynx software, with a noise reduction algorithm applied to remove peaks with a signal-to-noise ratio below 1,000 AU. The filtered spectra were then matched against protein databases.

### 2.10 Database mappings

Peptide sequences were determined from the molecular mass data of degradation products in the LC-MS spectra. The sample data were uploaded to a protein database search engine to identify the corresponding peptide sequences (31). The Swiss-Prot section of the UniProtKB database was used for peptide sequence identification (32). Allergenicity predictions were conducted using AllergenOnline.org, a database maintained by the Food Allergy Research and Resource Program at the University of Nebraska–Lincoln (33). The AllergenOnline database was searched using the “8-mer” option, enabling the detection of short-sequence peptides. Allergenicity potential was further evaluated using the AllerTOP v2.0 prediction tool, which applies multiple computational approaches, including machine learning algorithms (34, 35).

### 2.11 Statistical analysis

All experiments were conducted with at least three replicates, and the results were expressed as mean ± standard deviation (SD). The Chi-square test was used to analyze categorical data. For normally distributed continuous variables, one-way ANOVA followed by Tukey’s *post-hoc* test was applied to determine significant differences. Statistical significance was set at *p* < 0.05.

## 3 Results

**FIGURE 3:**
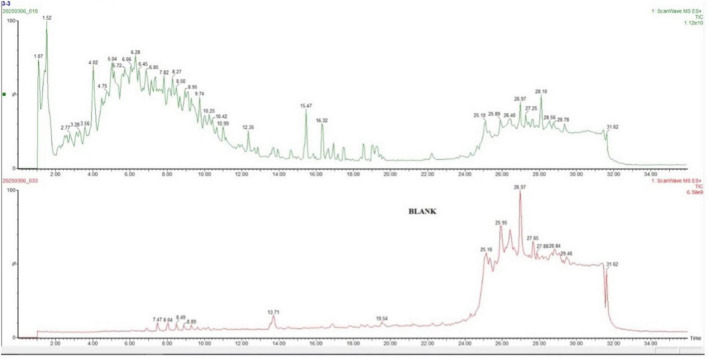
Total ion chromatogram.

To present the findings in a general-to-specific manner, total ion chromatograms (TICs) illustrating the overall profiles of two representative samples are shown in Figure 3. Figure 3 represents the *in vitro* digested sample subjected to microwave treatment. The figure displays the total ion signal of all compounds present in the sample. The chromatograms represent complex mixtures, with detected compounds presented in their raw form across the entire retention time. The microwave-cooked, *in vitro* digested sample exhibited a wide range of retention times between 1 and 34 min. In the Figure 3 shows the control (“blank”) sample consisted of *in vitro* digestion enzymes without the lupin substrate. In the blank sample, pronounced signals were observed after 24 min.

**FIGURE 4:**
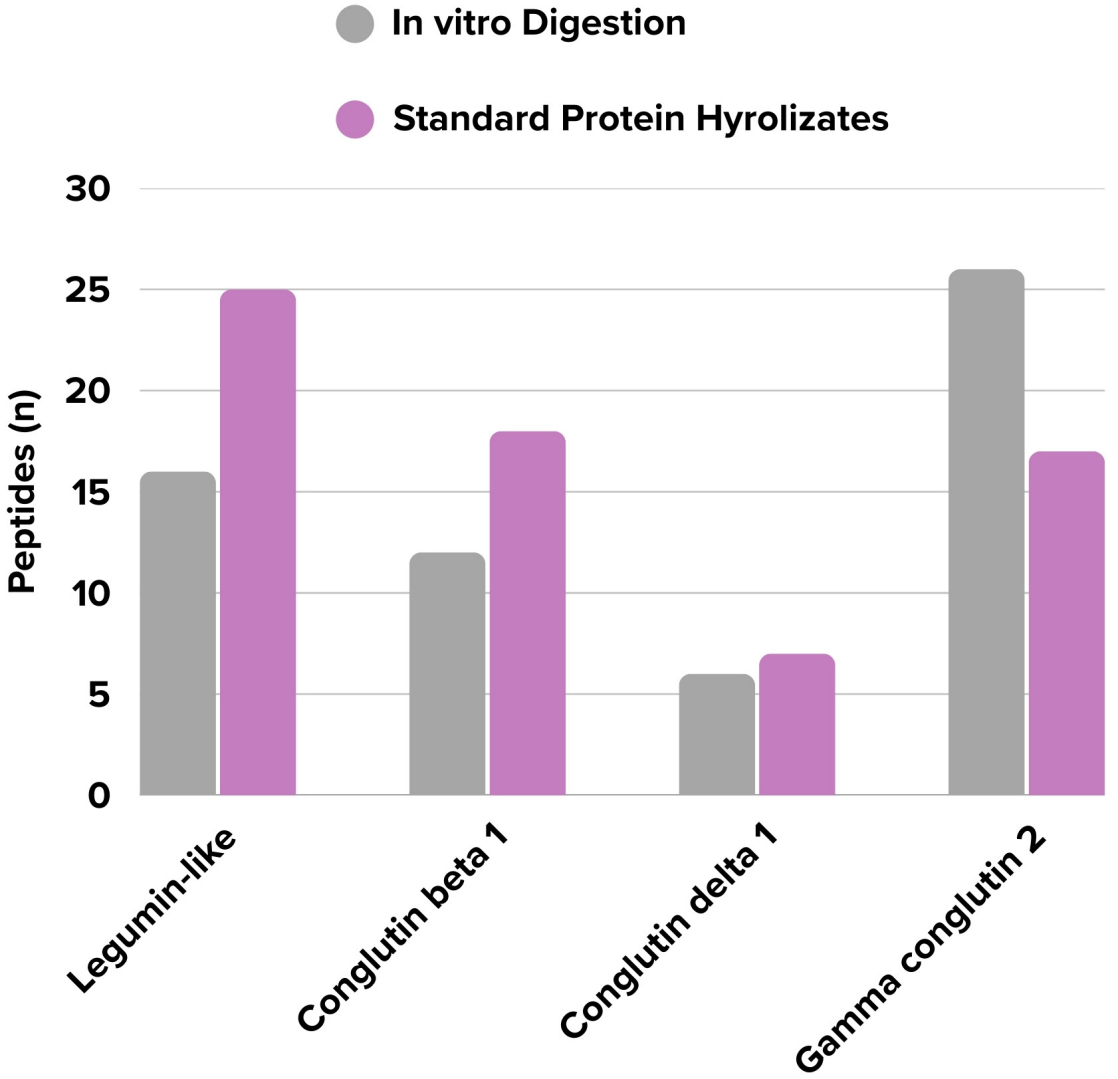
Comparison of the number of identified peptides by methods.

The distribution of the number of peptides identified peptide sequences *in vitro* digested and standard protein hydrolysates, based on their associated proteins, is presented in Figure 4. Although proteins from the same family were identified in both groups, notable differences peptide diversity were observed. In the *in vitro* digestion group, the number of distinct peptides belonging to Gamma conglutin 2 was higher (+9) compared to the undigested group. In contrast, fewer peptides (−9) from the Legumin-like protein were detected. This observation suggests a potential trend toward increased susceptibility of Gamma conglutin 2 to digestive enzymes. However, as no statistical significance was found for this difference (*p* > 0.05). the observed variation may reflect the inherent heterogeneity of the protein matrix. Despite this statistical limitation, the findings collectively indicate the selective nature of the *in vitro* digestion process on the lupin peptide profile.

**FIGURE 5:**
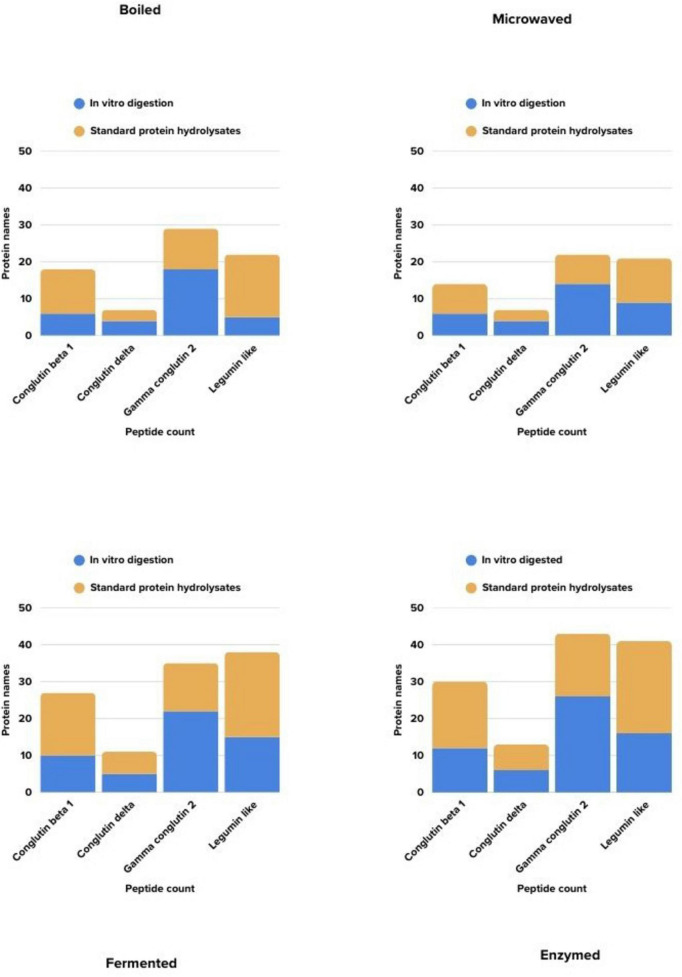
Comparison of the detected peptide numbers according to the methods.

Figure 5 compares the peptide diversity obtained from lupin by each cooking method, based on their associated proteins and whether they were subjected to *in vitro* gastrointestinal digestion. In the graph shown in a four-panel format, the blue bars represent the count of matched peptide sequences in samples that have undergone *in vitro* digestion, while the orange bars represent the number of unique sequences in samples that have not been subjected to *in vitro* digestion. Accordingly, some processes such as enzymes and fermentation cause increases in the diversity of detectable peptides after digestion, while in other methods, this increase has been more limited. These findings indicate that *in vitro* gastrointestinal digestion exerts method-specific effects on the peptide profile.

To compare the effects of different cooking methods, the protein–peptide matches for each method are summarized in Supplementary Tables 1–4. For instance, in the boiling method, 6 peptides were identified in the *in vitro* digestion sample of the conglutin beta 1 protein, while 12 peptides were identified in the non-digested samples. In the legumin-like protein and conglutin beta 1 protein, a higher diversity of peptides were detected in the samples without *in vitro* digestion, while in the conglutin delta protein, the sequence count was comparable, and in the gamma conglutin 2 protein, more peptides were detected in the sample with *in vitro* digestion. The *in vitro* digestion process exerted variable effects on the peptide profiles of proteins in the lupin samples.

*In silico* analyses were performed by matching the obtained peptide sequences with known allergen proteins in the AllergenOnline database. Complete matches were reported with their GenInfo Identifier (GI), along with the corresponding allergen protein and source organism (Supplementary Table 5). Most notably, a high frequency of matches was observed with the Conglutin beta isoforms (Lup an 1) of the species *Lupinus angustifolius*. These results indicates that the obtained peptides may contain IgE epitopes and could potentially pose an allergenic risk.

Supplementary Table 5 shows that for the samples subjected to *in vitro* digestion, some peptides we found to all four cooking methods match multiple allergenic proteins. For example, the peptides “YPSSTKDQ” and “TRYEEIQR” are the peptide sequences that showed the highest number of repeated matches in all four cooking methods in our samples. Similarly, peptides such as “KNRNGKIR” and “PKHSDADY” were detected only in enzymatic and fermentation cooking methods. The diversity of allergen matches in the boiling and microwave methods remained lower compared to the enzyme and fermentation methods. This situation suggests that enzymatic and fermentation processes more significantly affect and break down peptide sequences, thereby providing more opportunities for allergen matches.

Supplementary Table 6 summarizes the peptide sequences that showed complete matches with known allergen proteins in the AllergenOnline database. These matches are categorized according to cooking method and data from undigested samples. In all four different cooking methods, the peptide “PNTLILPK” matched multiple allergenic proteins. The legumin-like protein peptide “EEEEEEEE” was also detected in the boiling, enzymatic, and fermentation methods. In contrast, the “ILNPDDNQ” peptide from the conglutin beta 1 protein, which emerged only after enzyme and fermentation treatments, exhibited highly repetitive exact match. These methods promote for the further fragmentation of peptide structures, leading to the formation of short sequences with epitope similarity. Notably, in microwave-treated samples, the limited number of matching peptides suggests that these processes contribute less to the emergence of structures with allergenic potential.

Table 2 presents the results of peptide sequence matches with the AllerTop database, indicating their potential allergenicity. In the *in vitro* digested samples, four potential allergenic peptides from legumin-like protein, five from conglutin beta 1, four from conglutin delta, and five from gamma conglutin were identified. Additionally, peptides with allergenic potential that are non-allergenic generally have a sequence of 5 amino acids or fewer. In the standard protein hydrolysates method, eight potential allergenic peptides from legumin-like protein, three from conglutin beta 1, four from conglutin delta, and six from gamma conglutin were identified. These results indicate that the digestion process alters the predicted allergenic potential of the peptides. Only one overlapping allergenic peptide (QVVDCSGNAVF) was identified by both *in vitro* digestion and standard hydrolysis methods, indicating a method-dependent difference in allergen detection.

**TABLE 2:** Allergenicity potential of peptide sequences.

Protein name	Technique	Peptide sequence	Allergen status	Protein name	Technique	Peptide sequence	Allergen status
Legumin like protein	*İn vitro* digested	WMYNDGQTP VVAITL	Not allergen	Conglutin beta 1	*İn vitro* digested	YPSSTKDQQSYF	Allergen
		KEGDIIAVPTGIPF	Allergen			NTRYEEIQRIL	Allergen
		TIPQNYAAAIKSL	Not allergen			YKNRNGKIRVL	Allergen
		AAEHGSIYKNAL	Not allergen			RRQRNPYHF	Not allergen
		KTNDIPQIATL	Allergen			RIPAGSTSYIL	Not allergen
		QVVDCSGNAVF	Allergen			PKHSDADYVL	Allergen
		KNNNPYKF	Not allergen			QNYRIVEF	Allergen
		SGNQEQEF	Not allergen			DQRTNRL	Not allergen
		VPPPQSQL	Allergen			QSKPNTL	Not allergen
		EVVAHAF	Not allergen		Standard protein hydrolysates	QSKPNTLILPK	Not allergen
		NGSAWF	Not allergen			ILNPDDNQK	Not allergen
		IDTTNL	Not allergen			LENLQNY	Not allergen
	Standard Protein hydrolysates	NGELNEGQVLTI PQNY	Allergen			VLVVLNGR	Allergen
		EGGQGQQQEGG NVLSGF	Not allergen			IPAGSTSY	Not allergen
		EEEEEEEEEDER	Not allergen			NTLEATF	Allergen
		TLTSIDFPILGW	Not allergen			ILLGNED	Allergen
		QQPQENECQF	Allergen			HSDADY	Not allergen
		VIIPPTMRPR	Allergen			YPSSTK	Not allergen
		GMIFPGCGETY	Allergen	Conglutin delta	*İn vitro* digested	RRCNVNPDEE	Allergen
		QVVDCSGNAVF	Allergen			NSQRCQCRAL	Allergen
		LSGNQEQEF	Not allergen			DQCCEQL	Allergen
		NVNANSILY	Allergen			PRTCGF	Allergen
		EEPQESEK	Allergen		Standard protein hydrolysates	IQQQEEEEEGR	Not allergen
		TNAPQEIY	Allergen			ENQSEQCQGR	Allergen
		CAGVALSR	Not allergen			CNVNPDEE	Allergen
		NNNPYK	Not allergen			SSQQSCK	Allergen
Gamma conglutin	*İn vitro* digested	VMDSHDDVWRI SDENL	Allergen	Gamma conglutin	Standard protein hydrolysates	TPLMQVPVLL DLNGK	Allergen
		CHSTQCSRAN SHHCF	Allergen			IHNSIDVLIDMVY	Allergen
		YHNPQSTSSSSSKPSL	Not allergen			VMDSHDDVW	Allergen
		VDGGMHTRTEIVL	Not allergen			LSGGIPSVEF	Not allergen
		DNNYIHNSIDVL	Not allergen			VDGGMHTR	Allergen
		HWANIHKRTPL	Allergen			ANSHHCF	Allergen
		MSSNPVTQEAGF	Not allergen			DLDNNY	Allergen
		KSHGKTCANIF	Not allergen			AMCLSR	Not allergen
		MVQAQNGVSCL	Not allergen				
		RISQQGEYF	Allergen				
		AIHSTHGSKL	Not allergen				
		IDMVYTPL	Not allergen				
		ERSRVEF	Not allergen				
		SGGIPSVEF	Not allergen				
		EENMVVF	Allergen				
		PNNVQGPL	Allergen				
		GPMVRVL	Not allergen				
		MQVPVL	Not allergen				

## 4 Discussion

### 4.1 Chromatographic profile

In our study, the chromatographic profiles the samples exhibited peaks of varying intensities over a wide time range. A pronounced signal plateau was observed particularly after the 24th minute. In contrast, the chromatographic pattern of the blank sample differed considerably. In LC-MS/MS analysis, due to the reverse phase chromatography method used, short-chain hydrophilic peptides interact weakly with the column and are eluting earlier and generating peaks at shorter retention times, whereas more hydrophobic and larger peptides are retained longer on the column (36, 37). The initial peaks observed in the microwave-treated sample (between 4–15 min) are presumed to correspond predominantly shorter-chain, low molecular weight digestion products of peptides. In contrast, the peaks observed after the 24th minute likely represent larger peptide sequences, possibly derived from enzymatically specific cleavage sites. The observation of specific signals in the blank sample during this time frame suggests that the peptides may have formed through autolysis over time in the *in vitro* incubation period (38).

### 4.2 Peptide sequences

In our study, peptides belonging to the basic globular proteins of lupin were identified. Conglutin beta1 (β-conglutin); peptides components to the conglutin delta protein (δ-conglutin) and gamma conglutin 2 (γ-conglutin) have been identified. varying cooking methods have been shown to cause significant shifts in the sequences of detectable allergenic peptides. One notable finding was that *in vitro* digested samples (*n* = 60) yielded a lower diversity of detectable peptide sequences compared to undigested samples (*n* = 67). In a comprehensive study conducted on *Lupinus angustifolius*, the capture process of peptides with the highest peak signal intensity and the lowest technical variability for each conglutin protein was filtered for liquid chromatography multiple reaction monitoring-mass spectrometry (LC–MRM-MS). This approach identified 22, 37, 13, and 10 peptides from α-, β-, δ-, and γ-conglutin proteins, respectively, across 46 lupin genotypes (39).

Proteomic analyses have shown that lupin protein isolates are dominated by β-conglutin fragments, with numerous proteins involved in germination and stress responses identified in *Lupinus albus* (40–42). As indicated by these studies, the differences among the peptides identified in the literature can vary depending on the method used in the analysis (such as LC-MS/MS), the ionization energy settings, the mobile phase gradient protocol, the proteolytic enzymes applied during the sample preparation process, and the data processing parameters used for peptide identification. Such technical variables influence not only in the identified peptide sequences but also in the mass spectra of diversity of identified sequences (43, 44). Furthermore, most of the peptide sequences predicted by *in silico* analyzes were confirmed by LC-MS/MS after digestion supporting the predictive capability of the computational models. The absence of statistical significance in the differences between groups may be attributable to the limited sample size.

### 4.3 Impact of cooking methods on peptide profiles

Proteomic analyses on boiled beans revealed a decrease in the high molecular weight isoforms of the main storage globulin proteins, phaseolin (average change by −3.7-fold) and legumin (average change −2.5-fold) (45). In a study, low molecular weight peptides (< 3 kDa) were isolated from beans before and after grinding into flour and boiling. HPLC results showed greater peptide concentrations after the thermal treatment. Following the treatment, the bean peptides significantly decreased compared to the peptides obtained from native protein (46). In our study, the *in vitro* digestion process exerted different effects on the peptide profiles of the proteins found in lupin, cooked using the boiling method, depending on the protein family. One of the possible reasons for this observation is that the proteins in the *in vitro* gastrointestinal system were further degraded into smaller peptide sequences, rendering them undetectable by mass spectrometry (47). Indeed, in our study, the boiling process induced significant changes in the structure of lupin proteins, affecting their peptide profiles. Although each protein group responds differently, the general trend is that boiling breaks proteins into smaller fragments, enhancing enzyme accessibility during digestion.

Additionally, in another study, microwave cooking had the greatest impact on green peas and dry beans, while no thermal treatment significantly altered the peptide profile of chickpeas. Differences in the peptide profile depending on the type of legume and the applied thermal treatment were quantitatively demonstrated (48, 49). A combined approach has been proposed ultrasound and microwave techniques to obtain protein isolates from the seed of the *L. angustifolius* species grown in Ireland. SDS-PAGE analyses revealed that applying these processes preserved the primary structure of the proteins. However, high-power microwaves caused significant changes in the secondary structure of the proteins, transforming them from highly ordered forms (β-sheet and α-helix) to disordered forms (random coil and β-turn) (50).

The *in vitro* digestion model using the INFOGEST protocol enables the investigation of how proteins in processed foods are broken down in the gastrointestinal system, along with the resulting peptide profiles, and their biological activities in a laboratory setting (51). Consistent with the literature reporting increased protein digestibility and structural unfolding through microwave treatment of beans (52). Induces extensive denaturation of the protein structure, weakens intramolecular interactions, and thus unfold the protein chains, facilitating the access of digestive enzymes to the protein (53). In our study, conglutin delta showed an almost comparable peptide sequence diversity between treatments. In contrast, broader profile of unique peptides from gamma conglutin 2 were identified in the *in vitro* digested sample. In microwave-cooked samples, the *in vitro* digestion process exerted varying effects on the peptide profiles of the proteins found in lupin. It is likely that the partial denaturation of the protein or the formation of aggregates induced by thermal processing occurs differently depending on the lupin species (54).

The fermentation method, like other cooking methods, partially degrades proteins, but via a biological mechanism (19, 55, 56). During the fermentation of lupin seeds, proteases secreted by microorganisms (such as lactic acid bacteria) capable of degrading storage proteins, releasing smaller peptides and amino acids. It has also been concluded that the tannins, α-galactosides, phytic acid, and trypsin inhibitors of bacterial fermentation are also degraded (57, 58). Fermentation of *Lupinus angustifolius* flour has been suggested as a successful strategy to increase the acceptability of legume flavor (59, 60). Fermentation is known to degrade storage proteins and reduce allergenicity through microbial protease activity (61). Aligned with studies conducted on lupin, fermentation in alters protein content and quality in our study (62, 63). Tahmasian et al. (64) investigated the effect of fermentation induced by *Rhizopus oligosporus* on the proteome composition and allergenic protein content of *Lupinus albus* seeds. A significant reduction in the main *Lupinus albus* seeds allergen was reported, based on the peptide amount of the β-conglutin protein. Therefore, authors indicated that the fermentation process could be beneficial in reducing the potential allergenicity of lupin-based foods (64). The starter culture used in the current research was expected to consist of lactic acid bacteria derived from yogurt whey. Specific proteolytic cleavages carried out by microorganisms during fermentation may have contributed to the formation of these unique peptides. Additionally, the fermentation process may have induced covalent modifications in proteins (e.g., phosphorylation, deamidation), leading to differences in peptide masses, which could appear as new peaks in the LC-MS/MS profile. Consequently, these modifications may have led to the detection of different peptides. These findings support the view that the fermentation process is not only a traditional processing method but also a potential biotechnological tool for enhancing functional properties. A detailed investigation of the biological activities and health effects of peptides specifically identified in fermentation is warranted in future studies.

Enzymatic processes used as pre-treatments in foods represent a processing technology that can alter the protein structure and change their physicochemical properties. Such changes can be beneficial for formulating improved functional components and improving nutritional properties, adding value to legume proteins (65–67). Boschin et al. (68) showed that three types of lupin species exhibit similar activity scales, but after the application of pepsin + trypsin and chymotrypsin, *L. luteus* peptide mixtures have emerged as significantly the most active. In the study by Santos-Sánchez et al. (69), protein hydrolysate from *Lupinus angustifolius* was administered to mice, resulted in an increase in total plasma cholesterol, cardiovascular risk indices, and plasma antioxidant capacity in mice. Hydrolysates consisting of alcalase, papain, and pepsin were identified as the most effective for the degradation of α- and β-conglutinins, The enzymatic degradation of lupin has the potential to modify polypeptides that react with IgE and improve the techno-functional properties of the isolates, (70). In our study, the Gamma conglutin 2 protein was observed to have the most stable protein profile among all four different cooking methods examined. This protein largely maintained its structural integrity during the applied processes and produced thirteen peptides that were consistently identified under all conditions. This finding indicates that gamma conglutin 2 is structurally resistant to thermal treatments and other pre-treatments. Indeed, it has been reported in the literature that lupin seed proteins are highly resistant to thermal processes such as boiling or roasting (54). For legumin-like and conglutin beta 1 proteins, more peptides were detected in *in vitro* digestion, In conglutin delta, sequence counts were almost equal between treatments. In gamma conglutin 2, more peptides were detected in the *in vitro* digested sample. The *in vitro* digestion process, in addition to the enzyme treatment, exerted variable effects on the peptide profiles of the proteins found in lupin.

“YPSSTKDQ” and “TRYEEIQR” sequences matched in all four cooking methods. Their stability in all four cooking methods indicates that it is less affected by cooking processes and is highly conserved (71). The persistence of the specific peptide sequences YPSSTKDQ and TRYEEIQR across all four cooking methods warrants a molecular explanation regarding their resistance to proteolysis. The stability of YPSSTKDQ can be attributed to the presence of proline (P) at the second position. Proline is an imino acid with a rigid cyclic structure that imposes conformational constraints on the peptide backbone. It is well-documented that peptide bonds involving proline residues are often resistant to cleavage by broad-spectrum gastrointestinal proteases due to steric hindrance (72). Similarly, the resistance of the TRYEEIQR sequence is likely driven by its specific charge distribution. This sequence contains a cluster of acidic residues (Glutamic acid [E]) adjacent to bulky hydrophobic residues [tyrosine (Y), isoleucine (I)]. The high negative charge density created by the glutamic acid cluster can generate electrostatic repulsion or local structural folding that interferes with the binding efficiency of trypsin (73). Consequently, these molecular features proline-induced rigidity and charge-mediated shielding likely protect these epitopes from complete hydrolysis, preserving their structural integrity and potential IgE-binding capacity despite thermal and enzymatic processing.

In summary, comparative studies from the last decade have consistently shown that the use of these processes can transform lupin proteins. Each method has different effects but integrating them (e.g., optimizing the sequence) can often provide the most comprehensive improvements in lupin protein profiles and functionality (74, 75).

One study, the *in vitro* digestibility of certain legumes was assessed to determined that among all cooking methods, pressure cooking is the most effective (76). In another study, microwave cooking yields the highest levels of bioactive peptides after digestion in green peas and dried beans, while pressure cooking and traditional boiling were found to be less effective in terms of peptide diversity (48). In another study, autoclaving markedly increased the degree of sequential pepsin/pancreatin hydrolysis, whereas boiling increased it only in certain legumes (77).

### 4.4 Evaluation of methodological differences between *in vitro* digestion model and standard protein hydrolysates method approach

This study used a comparison of two models to evaluate the *in vivo* persistence potential of the identified epitopes. The INFOGEST protocol was used to simulate the complex, stepwise physiological conditions of human digestion, while a simplified protease assay was applied in parallel as a high-sensitivity biochemical control to test structural resistance. Consequently, peptide sequences demonstrating stability in both models may provide the strongest inference of resistance to proteolysis. As shown in Figure 4, although proteins from the same family were identified in both groups, notable differences in peptide diversity were observed, highlighting the selective nature of the physiological model. This divergence reconciles the perceived contradiction resolved by the various results shown in Figure 4 (an increase in some proteins and a decrease in others). Using a selective method like LC-MS/MS, this study demonstrated that the *in vitro* digestion significantly impacts the peptide profiles of proteins with effects varying by protein family.

In a study, the degradation of protein isolates obtained from various legumes and the peptide and free amino acid profiles reflecting the proteomic results under the INFOGEST protocol were analyzed. It was found that legumes, particularly soybeans, show resistance during gastric digestion, leading to the release of substantial amounts of peptides and amino acids (78). In lupin species, the closest study to this has investigated how different protein extraction methods of *Lupinus angustifolius* affect the proteome profile of lupin seeds. it demonstrates that the choice of method significantly affects the proteomic outcomes. This is crucial for understanding how digestion protocols can influence proteome comparisons (39, 79). To our knowledge, this is the first research conducted on *Lupinus albus*, which can compare the proteome profile obtained from the INFOGEST protocol with the standard protease method. *In vitro* digestion applied and unprocessed samples show that both peptide sequences belonging to the same protein (Conglutin beta 1) and those belonging to a different protein (legumin-like) matched exactly the allergen database. Although the same cooking methods were applied, treatment with different enzymes resulted in the formation of distinct peptide sequences in the lupin samples, which in turn produced different allergen matches. Additionally, the greater number of matches to known epitopes observed in samples subjected to an *in vitro* gastrointestinal system model suggests that this approach yields results more representative of allergenic peptides that may arise in a real biological environment.

### 4.5 Strengths and limitations of the study

Key strengths of this study include the use of high-resolution LC-MS/MS to determine the sequences of peptides formed after different cooking methods and *in vitro* digestion, as well as potential protein matches. This method is currently the most preferred technique in terms of sequence accuracy, precision, and comprehensiveness of data acquisition. As a cooking method, lactic acid bacteria fermentation is considered a relatively natural process that is safer than chemical/physical modification methods and can enrich the functional properties of legume proteins through biological degradation and modification. The use of lactic acid fermentation method in our study adds relevance to the work. Additionally, *in silico* analyses and bioinformatics databases were extensively utilized, providing detailed data and strengthening the analyses.

However, this study has certain limitations that should be acknowledged. First, the data presented relies on a label-free peptidomic approach, which provides semi-quantitative information based on peptide diversity and sequence counts rather than absolute quantification recommending future targeted studies for absolute quantification. Second the fact that the peptides providing the best match were identified using the LC-MS/MS method does not necessarily indicate the absence of other important protein-bound peptides; it only means that these peptides were not captured. Several factors could explain why these peptides were not captured. The peptide may have been degraded to the amino acid level, or it may have broken at a different point in the sequence. In addition, the ionization energies may not have been sufficient to detect these peptides. Furthermore, databases like Structural Database of Allergenic Proteins (SDAP) are less optimized for short peptide sequences, and some identified peptides did not meet the specific structural criteria of AllerTOP v2.0. Therefore, a definitive judgment cannot be made about the potential allergenicity of unassessed peptides. Nevertheless, peptides showing complete matches with *Lupinus* allergens in undigested samples (Supplementary Table 7) provide a valuable baseline for future risk assessment.

## 5 Conclusion

Our findings show that different cooking methods applied to lupin result in changes in the sequences of existing allergenic peptides. One notable finding is biotechnological methods (enzymatic treatment and fermentation) resulted in the most extensive modulation of the peptide profile, generating a wider array of detectable peptides compared to thermal treatments alone. A key observation was that *in vitro* digested samples contained fewer total peptide sequences compared to the standard technical control. This reduction does not imply a failure of detection; rather, it reflects the efficiency of the physiological digestion process in degrading susceptible protein regions into free amino acids or di-peptides, which are below the detection limit of peptidomics. Consequently, the peptides that were detected in the INFOGEST model represent the proteolytically resistant fraction. Furthermore, the survival of specific sequences in both the physiological model and the high-sensitivity control group suggests that their persistence stems from molecular stability rather than methodological errors. This confirms that the *in vitro* digestion model provides a more accurate representation of biological risk, as it filters out unstable fragments and selectively highlights the specific allergenic epitopes likely to persist in the human digestive tract. Our study provides substantial data that have the potential to guide future research. For example, the clinical effects of allergenic peptides identified in our study such as their interaction with human IgE antibodies could be evaluated using immunological methods like ELISA or Western blot. This would allow the observed trends to be examined from a different perspective. Examples from the literature demonstrate how *in vitro* gastrointestinal models help link specific processing conditions to peptide outcomes. By comparing such studies, a general trend emerges: efficient proteolysis can lead to the formation of a greater number of small bioactive peptides and fewer allergenic structures. This information can guide the food industry in developing legume-based products that maximize health benefits (e.g., antihypertensive, antioxidant peptides) while minimizing allergenic risk. Another suggestion for further research would be to include the effect of the microbiota in the INFOGEST protocol, which serves as the basis for *in vitro* bioavailability studies. Planning such a study would particularly provide an opportunity to deeply investigate and compare the changes observed in allergenic peptides through fermentation in our study and would complement our current findings.

In conclusion, this comprehensive study provides a robust, molecular-level foundation for understanding how domestic processing alters the allergenic risk of *Lupinus albus*. The data strongly support the use of optimized enzymatic or fermentation protocols as biotechnological tools to modulate peptide profiles, thereby maximizing protein bioaccessibility while providing a scientific basis for the food industry to develop safer, label-compliant, and functional legume-based products. However, despite processing, lupin retains allergenic characteristics. Therefore, it would be beneficial to impose a legal requirement for its labeling and to conduct regular inspections. The examination of the *in vivo* counterparts of these findings could shed light on future strategies to reduce lupin allergenicity.

## Data Availability

The datasets presented in this study can be found in online repositories. The names of the repository and accession number can be found below: https://doi.org/10.5281/zenodo.17774860.
