# Correction: Impact of thermal and biotechnological processing on the bioaccessibility and allergenic peptide profile of white lupin (*Lupinus albus*)

**DOI:** 10.3389/fnut.2026.1802774

**Published:** 2026-02-13

**Authors:** Fatmanur Ozyurek-Arpa, Mustafa Yaman, Bilal Çakir, Indrani Kalkan

**Affiliations:** 1 Department of Nutrition and Dietetics, Faculty of Health Sciences, Istanbul Medipol University, Istanbul, Türkiye; 2 Department of Nutrition and Dietetics, Institute of Health Sciences, Istanbul Medipol University, Istanbul, Türkiye; 3 Department of Molecular Biology and Genetics, Faculty of Engineering and Natural Sciences, Istanbul Sabahattin Zaim University, Istanbul, Türkiye; 4 Halal Food R&D Center, Sabri Ülker R&D Center Bldg, Istanbul S. Zaim University (IZÜ), Istanbul, Türkiye; 5 IZÜ Food and Agricultural Research Center (GTUAM), Halkali Campus, Istanbul, Türkiye

**Keywords:** allergenicity, bioaccessibility, *in vitro* digestion, *Lupinus albus*, peptide, sequence

In the published article, there was an error regarding the Correspondence section. Author Indrani Kalkan was erroneously omitted as a co-corresponding author. The correct corresponding authors are Fatmanur Ozyurek-Arpa and Indrani Kalkan.

The original version of this article has been updated.

