# Supplementary material for: Impact of thermal and biotechnological processing on the bioaccessibility and allergenic peptide profile of white lupin (Lupinus albus)
Source: Front Nutr. 2026 Jan 28;12:1757989. doi: 10.3389/fnut.2025.1757989 (PMC12893724; doi:10.3389/fnut.2025.1757989)
Supplement: Supplementary file 1 [file Table_1.docx]

**Supplementary Table 1. Protein and peptide matches by boiling method according to digestion groups**

| **Protein names** | **Digestion Groups (Peptide Count)** | **Peptide Sequences** | **Protein names** | **Digestion Groups (Peptide Count)** | **Peptide Sequences** | **Protein names** | **Digestion Groups (Peptide Count)** | **Peptide Sequences** |
| --- | --- | --- | --- | --- | --- | --- | --- | --- |
| Conglutin beta 1 | *In vitro digestion* (6) | YPSSTKDQQSYF | Gamma conglutin 2 | *In vitro digestion* (18) | VMDSHDDVWRISDENL | Legumin-like | *In vitro digestion* (5) | WMYNDGQTPVVAITL |
|  |  | NTRYEEIQRIL |  |  | CHSTQCSRANSHHCF |  |  | KEGDIIAVPTGIPF |
|  |  | PKHSDADYVL |  |  | YHNPQSTSSSSSKPSL |  |  | KTNDIPQIATL |
|  |  | QNYRIVEF |  |  | VDGGMHTRTEIVL |  |  | NGSAWF |
|  |  | DQRTNRL |  |  | DNNYIHNSIDVL |  |  | IDTTNL |
|  |  | QSKPNTL |  |  | HWANIHKRTPL |  | *Standard protein hydrolysates* (17) | NGELNEGQVLTIPQNY |
|  | *Standard protein hydrolysates* (12) | QSKPNTLILPK |  |  | MSSNPVTQEAGF |  |  | EGGQGQQQEGGNVLSGF |
|  |  | LENLQNY |  |  | KSHGKTCANIF |  |  | EEEEEEEEEDER |
|  |  | IPAGSTSY |  |  | AIHSTHGSKL |  |  | TLTSIDFPILGW |
|  |  | NTLEATF |  |  | IDMVYTPL |  |  | VIIPPTMRPR |
|  |  | ILLGNED |  |  | ERSRVEF |  |  | GMIFPGCGETY |
|  |  | HSDADY |  |  | PNNVQGPL |  |  | LSGNQEQEF |
|  |  | YPSSTK |  |  | GPMVRVL |  |  | NVNANSILY |
|  |  | NPYHF |  |  | MQVPVL |  |  | TNAPQEIY |
|  |  | EEIQR |  |  | HHSIF |  |  | CAGVALSR |
|  |  | DQQSY |  |  | SCAPSF |  |  | NNNPYK |
|  |  | NLEY |  |  | KRQF |  |  | HHHHR |
|  |  | GDALR |  |  | AQDVL |  |  | SLSDNF |
| Conglutin delta | *In vitro digestion* (4) | RRCNVNPDEE |  | *Standard protein hydrolysates* (11) | TPLMQVPVLLDLNGK |  |  | NDELR |
|  |  | NSQRCQCRAL |  |  | IHNSIDVLIDMVY |  |  | SHQVR |
|  |  | PRTCGF |  |  | LSGGIPSVEF |  |  | IQQGR |
|  |  | DQCCEQL |  |  | VDGGMHTR |  |  | HHHHR |
|  | *Standard protein hydrolysates* (3) | IQQQEEEEEGR |  |  | DLDNNY |  |  |  |
|  |  | ENQSEQCQGR |  |  | AMCLSR |  |  |  |
|  |  | CNVNPDEE |  |  | NSNSLK |  |  |  |
|  |  |  |  |  | SCAPSF |  |  |  |
|  |  |  |  |  | ANIHK |  |  |  |
|  |  |  |  |  | VTCSY |  |  |  |
|  |  |  |  |  | HGDSR |  |  |  |

**Supplementary Table 2. Protein and peptide matches by microwave method according to digestion groups**

| **Protein names** | **Digestion Groups (Peptide Count)** | **Peptide Sequences** | **Protein names** | **Digestion Groups (Peptide Count)** | **Peptide Sequences** | **Protein names** | **Digestion Groups (Peptide Count)** | **Peptide Sequences** |
| --- | --- | --- | --- | --- | --- | --- | --- | --- |
| Conglutin beta 1 | *In vitro digestion* (6) | YPSSTKDQQSYF | Gamma conglutin 2 | *In vitro digestion* (14) | VMDSHDDVWRISDENL | Legumin-like | *In vitro digestion* (9) | WMYNDGQTPVVAITL |
|  |  | NTRYEEIQRIL |  |  | CHSTQCSRANSHHCF |  |  | KEGDIIAVPTGIPF |
|  |  | PKHSDADYVL |  |  | YHNPQSTSSSSSKPSL |  |  | KTNDIPQIATL |
|  |  | QNYRIVEF |  |  | DNNYIHNSIDVL |  |  | QVVDCSGNAVF |
|  |  | DQRTNRL |  |  | MSSNPVTQEAGF |  |  | KNNNPYKF |
|  |  | QSKPNTL |  |  | KSHGKTCANIF |  |  | SGNQEQEF |
|  | *Standard protein hydrolysates* (8) | QSKPNTLILPK |  |  | AIHSTHGSKL |  |  | VPPPQSQL |
|  |  | LENLQNY |  |  | IDMVYTPL |  |  | CTMKL |
|  |  | ILLGNED |  |  | ERSRVEF |  |  | IDTTNL |
|  |  | HSDADY |  |  | PNNVQGPL |  | *Standard protein hydrolysates*(12) | NGELNEGQVLTIPQNY |
|  |  | YPSSTK |  |  | MQVPVL |  |  | EGGQGQQQEGGNVLSGF |
|  |  | EEIQR |  |  | SCAPSF |  |  | TLTSIDFPILGW |
|  |  | DQQSY |  |  | KRQF |  |  | VIIPPTMRPR |
|  |  | GDALR |  |  | AQDVL |  |  | GMIFPGCGETY |
| Conglutin delta | *In vitro digestion* (4) | RRCNVNPDEE |  | *Standard protein hydrolysates* (8) | TPLMQVPVLLDLNGK |  |  | LSGNQEQEF |
|  |  | NSQRCQCRAL |  |  | IHNSIDVLIDMVY |  |  | NVNANSILY |
|  |  | PRTCGF |  |  | LSGGIPSVEF |  |  | TNAPQEIY |
|  |  | DQCCEQL |  |  | VDGGMHTR |  |  | CAGVALSR |
|  | *Standard protein hydrolysates* (3) | IQQQEEEEEGR |  |  | DLDNNY |  |  | NNNPYK |
|  |  | ENQSEQCQGR |  |  | AMCLSR |  |  | NDELR |
|  |  | CNVNPDEE |  |  | VTCSY |  |  | IQQGR |
|  |  |  |  |  | HGDSR |  |  |  |

**Supplementary Table 3. Protein and peptide matches by fermentation method according to digestion groups**

| **Protein names** | **Digestion Groups (Peptide Count)** | **Peptide Sequences** | **Protein names** | **Digestion Groups (Peptide Count)** | **Peptide Sequences** | **Protein names** | **Digestion Groups (Peptide Count)** | | **Peptide Sequences** |
| --- | --- | --- | --- | --- | --- | --- | --- | --- | --- |
| Conglutin beta 1 | *In vitro digestion* (10) | YPSSTKDQQSYF | Conglutin delta | *In vitro digestion* (5) | RRCNVNPDEE | Gamma conglutin 2 |  | | MQVPVL |
|  |  | NTRYEEIQRIL |  |  | NSQRCQCRAL |  |  |  | HHSIF |
|  |  | YKNRNGKIRVL |  |  | PRTCGF |  |  |  | SCAPSF |
|  |  | RRQRNPYHF |  |  | QQVNL |  |  |  | KRQF |
|  |  | RIPAGSTSYIL |  |  | EQEL |  |  |  | GTHQL |
|  |  | PKHSDADYVL |  | *Standard protein hydrolysates* (6) | IQQQEEEEEGR |  |  |  | AQDVL |
|  |  | QSKPNTL |  |  | CNVNPDEE |  |  |  | NSNSL |
|  |  | NSQRF |  |  | SSQQSCK |  |  |  | AQKGL |
|  |  | RVVKL |  |  | ALQQIY |  |  |  | QNQL |
|  |  | SRNTL |  |  | HVIR |  | *Standard protein hydrolysates* (13) | | TPLMQVPVLLDLNGK |
|  | *Standard protein hydrolysates* (17) | QSKPNTLILPK |  |  | CQCR |  |  |  | VMDSHDDVW |
|  |  | ILNPDDNQK | Gamma conglutin 2 | *In vitro digestion* (22) | VMDSHDDVWRISDENL |  |  |  | LSGGIPSVEF |
|  |  | LENLQNY |  |  | CHSTQCSRANSHHCF |  |  |  | ANSHHCF |
|  |  | VLVVLNGR |  |  | YHNPQSTSSSSSKPSL |  |  |  | DLDNNY |
|  |  | IPAGSTSY |  |  | VDGGMHTRTEIVL |  |  |  | NSNSLK |
|  |  | NTLEATF |  |  | DNNYIHNSIDVL |  |  |  | SCAPSF |
|  |  | ILLGNED |  |  | HWANIHKRTPL |  |  |  | ANIHK |
|  |  | YPSSTK |  |  | MSSNPVTQEAGF |  |  |  | VTCSY |
|  |  | NPYHF |  |  | KSHGKTCANIF |  |  |  | DLNNA |
|  |  | EEIQR |  |  | MVQAQNGVSCL |  |  |  | SSSTY |
|  |  | DQQSY |  |  | RISQQGEYF |  |  |  | DLER |
|  |  | NLEY |  |  | AIHSTHGSKL |  |  |  | VLQY |
|  |  | GDALR |  |  | ERSRVEF |  |  | |  |
|  |  | QTLY |  |  | SGGIPSVEF |  |  | |  |
|  |  | VLER |  |  | EENMVVF |  |  | |  |
|  |  | IVEF |  |  | PNNVQGPL |  |  | |  |
|  |  | NSQR |  |  | GPMVRVL |  |  | |  |
|  |  |  |  |  |  |  |  | |  |
|  |  |  |  |  |  |  |  | |  |
|  |  |  |  |  |  |  |  | |  |
| **Protein names** | **Digestion Groups (Peptide Count)** | **Peptide Sequences** | **Protein names** | **Digestion Groups (Peptide Count)** | **Peptide Sequences** |  |  |  | |
| Legumin-like | *In vitro digestion* (15) | WMYNDGQTPVVAITL |  |  | NNNPYK |  |  |  | |
|  |  | KEGDIIAVPTGIPF |  |  | HHHHR |  |  |  | |
|  |  | TIPQNYAAAIKSL |  |  | SLSDNF |  |  |  | |
|  |  | AAEHGSIYKNAL |  |  | NDELR |  |  |  | |
|  |  | KTNDIPQIATL |  |  | SHQVR |  |  |  | |
|  |  | QVVDCSGNAVF |  |  | IQQGR |  |  |  | |
|  |  | KNNNPYKF |  |  | RPHR |  |  |  | |
|  |  | SGNQEQEF |  |  | VPYY |  |  |  | |
|  |  | VPPPQSQL |  |  | VEHF |  |  |  | |
|  |  | EVVAHAF |  |  | DDEF |  |  |  | |
|  |  | NGSAWF |  |  | NLNR |  |  |  | |
|  |  | RYVAF |  |  |  |  |  |  | |
|  |  | CTMKL |  |  |  |  |  |  | |
|  |  | RRPF |  |  |  |  |  |  | |
|  |  | DDEF |  |  |  |  |  |  | |
|  | *Standard protein hydrolysates* (23) | NGELNEGQVLTIPQNY |  |  |  |  |  |  | |
|  |  | EEEEEEEEEDER |  |  |  |  |  |  | |
|  |  | TLTSIDFPILGW |  |  |  |  |  |  | |
|  |  | QQPQENECQF |  |  |  |  |  |  | |
|  |  | VIIPPTMRPR |  |  |  |  |  |  | |
|  |  | GMIFPGCGETY |  |  |  |  |  |  | |
|  |  | QVVDCSGNAVF |  |  |  |  |  |  | |
|  |  | LSGNQEQEF |  |  |  |  |  |  | |
|  |  | NVNANSILY |  |  |  |  |  |  | |
|  |  | EEPQESEK |  |  |  |  |  |  | |
|  |  | TNAPQEIY |  |  |  |  |  |  | |
|  |  | EGGIVEVK |  |  |  |  |  |  | |
|  |  | CAGVALSR |  |  |  |  |  |  | |
|  |  |  |  |  |  |  |  |  | |

**Supplementary Table 4. Protein and peptide matches by enzyme method according to digestion groups**

| **Protein names** | **Digestion Groups (Peptide Count)** | **Peptide Sequences** | **Protein names** | **Digestion Groups (Peptide Count)** | **Peptide Sequences** | **Protein names** | **Digestion Groups (Peptide Count)** | **Peptide Sequences** |
| --- | --- | --- | --- | --- | --- | --- | --- | --- |
| Conglutin beta 1 | *In vitro digestion* (12) | YPSSTKDQQSYF | Conglutin delta | *In vitro digestion* (6) | RRCNVNPDEE | Gamma conglutin 2 | *In vitro digestion* (26) | MQVPVL |
|  |  | NTRYEEIQRIL |  |  | NSQRCQCRAL |  |  | HHSIF |
|  |  | PKHSDADYVL |  |  | DQCCEQL |  |  | SCAPSF |
|  |  | QSKPNTL |  |  | PRTCGF |  |  | KRQF |
|  |  | QNYRIVEF |  |  | QQVNL |  |  | GTHQL |
|  |  | DQRTNRL |  |  | DQCCEQL |  |  | AQDVL |
|  |  | YKNRNGKIRVL |  | *Standard protein hydrolysates* (7) | IQQQEEEEEGR |  |  | NSNSL |
|  |  | RRQRNPYHF |  |  | ENQSEQCQGR |  |  | AQKGL |
|  |  | RIPAGSTSYIL |  |  | CNVNPDEE |  |  | QNQL |
|  |  | NSQRF |  |  | SSQQSCK |  | *Standard protein hydrolysates* (17) | TPLMQVPVLLDLNGK |
|  |  | RVVKL |  |  | ALQQIY |  |  | IHNSIDVLIDMVY |
|  |  | SRNTL |  |  | HVIR |  |  | VMDSHDDVW |
|  | *Standard protein hydrolysates* (18) | QSKPNTLILPK |  |  | CQCR |  |  | LSGGIPSVEF |
|  |  | ILNPDDNQK | Gamma conglutin 2 | *In vitro digestion* (26) | VMDSHDDVWRISDENL |  |  | VDGGMHTR |
|  |  | LENLQNY |  |  | CHSTQCSRANSHHCF |  |  | ANSHHCF |
|  |  | VLVVLNGR |  |  | YHNPQSTSSSSSKPSL |  |  | DLDNNY |
|  |  | IPAGSTSY |  |  | VDGGMHTRTEIVL |  |  | AMCLSR |
|  |  | NTLEATF |  |  | DNNYIHNSIDVL |  |  | NSNSLK |
|  |  | ILLGNED |  |  | HWANIHKRTPL |  |  | SCAPSF |
|  |  | HSDADY |  |  | MSSNPVTQEAGF |  |  | ANIHK |
|  |  | YPSSTK |  |  | KSHGKTCANIF |  |  | VTCSY |
|  |  | NPYHF |  |  | MVQAQNGVSCL |  |  | HGDSR |
|  |  | EEIQR |  |  | RISQQGEYF |  |  | DLNNA |
|  |  | DQQSY |  |  | AIHSTHGSKL |  |  | SSSTY |
|  |  | NLEY |  |  | IDMVYTPL |  |  | DLER |
|  |  | GDALR |  |  | ERSRVEF |  |  | VLQY |
|  |  | QTLY |  |  | SGGIPSVEF |  |  |  |
|  |  | VLER |  |  | EENMVVF |  |  |  |
|  |  | IVEF |  |  | PNNVQGPL |  |  |  |
|  |  | NSQR |  |  | GPMVRVL |  |  |  |
| **Protein names** | **Digestion Groups (Peptide Count)** | **Peptide Sequences** | **Protein names** | **Digestion Groups (Peptide Count)** | **Peptide Sequences** |  |  |  |
| Legumin-like | *In vitro digestion* (16) | WMYNDGQTPVVAITL | Legumin-like | *Standard protein hydrolysates* (25) | EGGIVEVK |  |  |  |
|  |  | KEGDIIAVPTGIPF |  |  | CAGVALSR |  |  |  |
|  |  | KTNDIPQIATL |  |  | NNNPYK |  |  |  |
|  |  | QVVDCSGNAVF |  |  | HHHHR |  |  |  |
|  |  | KNNNPYKF |  |  | SLSDNF |  |  |  |
|  |  | SGNQEQEF |  |  | NDELR |  |  |  |
|  |  | VPPPQSQL |  |  | SHQVR |  |  |  |
|  |  | CTMKL |  |  | IQQGR |  |  |  |
|  |  | NGSAWF |  |  | RPHR |  |  |  |
|  |  | IDTTNL |  |  | VPYY |  |  |  |
|  |  | TIPQNYAAAIKSL |  |  | VEHF |  |  |  |
|  |  | AAEHGSIYKNAL |  |  | DDEF |  |  |  |
|  |  | EVVAHAF |  |  | NLNR |  |  |  |
|  |  | RYVAF |  |  |  |  |  |  |
|  |  | RRPF |  |  |  |  |  |  |
|  |  | DDEF |  |  |  |  |  |  |
|  | *Standard protein hydrolysates* (25) | NGELNEGQVLTIPQNY |  |  |  |  |  |  |
|  |  | EGGQGQQQEGGNVLSGF |  |  |  |  |  |  |
|  |  | EEEEEEEEEDER |  |  |  |  |  |  |
|  |  | TLTSIDFPILGW |  |  |  |  |  |  |
|  |  | QQPQENECQF |  |  |  |  |  |  |
|  |  | VIIPPTMRPR |  |  |  |  |  |  |
|  |  | GMIFPGCGETY |  |  |  |  |  |  |
|  |  | QVVDCSGNAVF |  |  |  |  |  |  |
|  |  | LSGNQEQEF |  |  |  |  |  |  |
|  |  | NVNANSILY |  |  |  |  |  |  |
|  |  | EEPQESEK |  |  |  |  |  |  |
|  |  |  |  |  |  |  |  |  |

**Supplementary Table 5. Allergenicity database matching results of in vitro digested samples according to cooking methods**

| **Cooking methods** | **Peptide sequence** | **GI no** | **Matched allergen protein** | **Origin** |
| --- | --- | --- | --- | --- |
| Boiling, Microwave, Enzyme, Fermentation  (Conglutin beta 1) | YPSSTKDQ | 149208401  980951568  980951548  89994190  75121065 | Conglutin beta 7 (Lup an 1)  Conglutin beta 2 (Lup an 1)  Vicilin-like protein,  Conglutin beta 2 | *Lupinus angustifolius,*  *Lupinus albus* |
|  | PSSTKDQQ |  |  |  |
|  | SSTKDQQS |  |  |  |
|  | STKDQQSY |  |  |  |
|  | TKDQQSYF |  |  |  |
|  | TKDQQSYF | 149208403  169950562  980951561  980951518 | Lup an 1 (bazıları) | *Lupinus angustifolius* |
| Boiling, Microwave, Enzyme, Fermentation  (Conglutin beta 1) | NTRYEEIQ | 149208401  980951568  980951565  980951555  89994190 | Conglutin beta  Conglutin beta 7 (Lup an 1)  Conglutin beta 6 (Lup an 1)  Conglutin beta 4 (Lup an 1)  Vicilin-like protein, | *Lupinus angustifolius*  *Lupinus albus* |
|  | TRYEEIQR |  |  |  |
|  | RYEEIQRI |  |  |  |
|  | YEEIQRIL |  |  |  |
|  | NTRYEEIQ | 980951550 | Conglutin beta 3 (Lup an 1) | *Lupinus angustifolius* |
|  | NTRYEEIQ | 980951548  75121065 | Conglutin beta 3 (Lup an 1)  Conglutin beta 2(Lup an 1) | *Lupinus angustifolius* |
|  | TRYEEIQR |  |  |  |
|  | RYEEIQRI |  |  |  |
|  | YEEIQRIL | 980951561 | Conglutin beta 5 (Lup an 1) | *Lupinus angustifolius* |
| Enzyme, Fermentation (Conglutin beta 1) | YKNRNGKI | 89994190  75121065 | Vicilin-like protein  Conglutin beta 2(Lup an 1) | *Lupinus albus*  *Lupinus angustifolius* |
|  | KNRNGKIR |  |  |  |
|  | NRNGKIRV |  |  |  |
|  | RNGKIRVL |  |  |  |
| Enzyme, Fermentation (Conglutin beta 1) | RRQRNPYH | 149208401  149208403  169950562  980951568  89994190  75121065 | Conglutin beta (Lup an 1) | *Lupinus angustifolius* |
|  | RQRNPYHF |  | Conglutin beta 7 (Lup an 1)  Conglutin beta 1 (Lup an 1)  Vicilin-like protein  Conglutin beta 2(Lup an 1) | *Lupinus albus* |
|  | RIPAGSTS | 89994190  75121065 | Vicilin-like protein  Conglutin beta 2(Lup an 1) | *Lupinus albus*  *Lupinus angustifolius* |
|  | IPAGSTSY |  |  |  |
|  | PAGSTSYI |  |  |  |
|  | AGSTSYIL |  |  |  |
| Enzyme, Fermentation (Conglutin beta 1) | PKHSDADY | 149208401  980951568  980951565  980951561  980951555  980951550  980951548 | Conglutin beta (Lup an 1)  Conglutin beta 7 (Lup an 1)  Conglutin beta 6 (Lup an 1)  Conglutin beta 5 (Lup an 1)  Conglutin beta 4 (Lup an 1)  Conglutin beta 3 (Lup an 1)  Conglutin beta 2 (Lup an 1) | *Lupinus angustifolius* |
|  | PKHSDADY | 89994190  75121065 | Vicilin-like protein  Conglutin beta 2 (Lup an 1) | *Lupinus albus*  *Lupinus angustifolius* |
|  | KHSDADYV |  |  |  |
|  | HSDADYVL |  |  |  |
| Boiling, Microwave, Enzyme  (Conglutin beta 1) | QNYRIVEF | 149208401  980951568  980951565  980951561  980951555  980951550  980951548  89994190  75121065 | Conglutin beta (Lup an 1)  Conglutin beta 7 (Lup an 1)  Conglutin beta 6 (Lup an 1)  Conglutin beta 5 (Lup an 1)  Conglutin beta 4 (Lup an 1)  Conglutin beta 3 (Lup an 1)  Conglutin beta 2 (Lup an 1)  Conglutin beta 2 (Lup an 1)  Vicilin-like protein  Conglutin beta 2 (Lup an 1) | *Lupinus angustifolius* |

**Supplementary Table 6. Matching of peptide sequences to allergenic epitopes in standard protein hydrolysates samples**

| **Cooking methods** | **Peptide sequence** | **GI no** | **Matched allergen protein** | **Origin** |
| --- | --- | --- | --- | --- |
| Boiling, Enzyme, Fermentation  (Legumin like) | EEEEEEEE | 149208401  149208403  169950562  980951568  980951518 | Conglutin beta (Lup an 1)  Conglutin beta 7 (Lup an 1)  Conglutin beta 1 (Lup an 1) | *Lupinus angustifolius* |
| Boiling, Microwave, Enzyme, Fermentation  (Conglutin beta 1) | QSKPNTLI | 149208403  169950562  980951565  980951555  980951550  980951548  980951518  89994190  75121065 | Conglutin beta (Lup an 1)  Conglutin beta 6 (Lup an 1)  Conglutin beta 4 (Lup an 1)  Conglutin beta 3 (Lup an 1)  Conglutin beta 2 (Lup an 1)  Conglutin beta 1 (Lup an 1)  Vicilin-like protein | *Lupinus angustifolius*  *Lupinus albus* |
|  | SKPNTLIL |  |  |  |
|  | KPNTLILP |  |  |  |
|  | PNTLILPK |  |  |  |
|  | PNTLILPK | 149208401  980951568  980951561 | Conglutin beta (Lup an 1)  Conglutin beta 7 (Lup an 1)  Conglutin beta 5 (Lup an 1) | *Lupinus angustifolius* |
| Enzyme, Fermentation  (Conglutin beta 1) | ILNPDDNQ | 149208401  149208403  169950562  980951568  980951565  980951561  980951555  980951550  980951548  980951518 | Conglutin beta (Lup an 1)  Conglutin beta 6 (Lup an 1)  Conglutin beta 4 (Lup an 1)  Conglutin beta 3 (Lup an 1)  Conglutin beta 2 (Lup an 1)  Conglutin beta 1 (Lup an 1) | *Lupinus angustifolius* |
|  | ILNPDDNQ | 89994190  75121065 | Vicilin-like protein  Conglutin beta 2 (Lup an 1) | *Lupinus angustifolius*  *Lupinus albus* |
|  | LNPDDNQK |  |  |  |
| Enzyme, Fermentation  (Conglutin beta 1) | VLVVLNGR | 89994190  75121065 | Vicilin-like protein  Conglutin beta 2 (Lup an 1) | *Lupinus angustifolius*  *Lupinus albus* |
| Boiling, Enzyme, Fermentation | IPAGSTSY | 89994190  75121065 | Vicilin-like protein  Conglutin beta 2 (Lup an 1) | *Lupinus angustifolius*  *Lupinus albus* |

Non-Available

**Supplementary Table 6: Allergen matches with organisms other than Lupin species in samples not subjected to in vitro gastrointestinal tract**

| **Pişirme Yöntemi** | **Peptit Sekansı** | **GI numarası** | **Eşleştiği alerjen protein** | **Kaynak organizma** |
| --- | --- | --- | --- | --- |
| Boiling, Microwave, Enzyme, Fermentation | GQVLTIPQ | 18479082  557792009 | 11S globulin-like protein  Cor a 9 allergen | *Corylus avellana* |
|  | QVLTIPQN |  |  |  |
| Boiling, Microwave, Enzyme, | EGGQGQQQ | 523916668 | putative Pru du 6 allergen, | *Prunus dulcis* |
|  |  | 307159114 | prunin 2 precursor |  |
| Boiling, Enzyme, Fermentation | EEEEEEED | 18615  169973 | Glycinin-like  glycinin A-1a-B-x subunit | *Glycine max* |
|  | EEEEEEDE |  |  |  |
| Boiling, Microwave, Enzyme, Fermentation | TNAPQEIY | 18639 | glycinin subunit G3 | *Glycine max* |
| Boiling, Microwave, Enzyme, Fermentation | CAGVALSR | 18639  218265  18609  169973  18615  312233065  224036293  199732457  22135348  112380623  21314465  5712199  3703107 | glycinin subunit G3  glycinin A2B1a subunit  glycinin A-1a-B-x subunit  Glycinin-like  Ara h 3 allergen  Chain A, Crystal Structure Of Peanut Major Allergen Ara H 3  arachin Arah3 isoform  iso-Ara h3  trypsin inhibitor  allergen Arah3/Arah4  glycinin | *Glycine max*  *Arachis hypogaea* |
| Boiling, Microwave, Enzyme, Fermentation | QQEEEEEG | 18641  732706  806556  4249568 | Glycinin  Glycinin-like  A5A4B3 subunit | *Glycine max*  *Glycine soja* |
